# Comparing age and sex trends of chlamydia, gonorrhoea, hepatitis and syphilis infections in Samoa in 2012 and 2017

**DOI:** 10.5365/wpsar.2019.10.2.004

**Published:** 2020-03-31

**Authors:** Robert Carney, Michaela Howells, Aaone Tanumafili, Athena Matalavea, Judith Gafa, Dr Take Naseri Leausa Toleafoa

**Affiliations:** a Samoa Ministry of Health, Apia, Samoa.; b Department of Anthropology, University of North Carolina, Wilmington, North Carolina, United States of America.

## Abstract

In Samoa, the seroprevalence rates of sexually transmitted infections other than HIV have been endemically high over the past decade, despite years of prevention programming. Odds ratio and χ^2^ tests were conducted to compare the rates of positivity of chlamydia, gonorrhoea, hepatitis B and C, and syphilis across age groups from 2012 and 2017 surveillance data in Samoa. Young people aged 15–19 years were significantly more likely to have a chlamydia infection compared to all other age groups in both 2012 and 2017. Hepatitis B infections were more common in males and those aged 30 and above in both 2012 and 2017. Hepatitis C had no significant differences in age, but it was more common in males in 2012 and more common in females in 2017. Older age groups (aged 45 and above) were more likely to have a positive syphilis test in both 2014 and 2017 when compared to those aged 15–24 years. The results of this analysis confirm previously observed trends in Samoa for younger age groups’ prevalence of chlamydia and gonorrhoea, and for older age groups’ prevalence of hepatitis B and C. But the analysis also unexpectedly found that older age groups (aged 45 and above) are more likely to test positive for syphilis (for years 2014 and 2017). Further studies are needed to assess behavioural risk factors associated with older populations to explain the increase in risk and to design interventions suited to this demographic.

Sexually transmitted infections (STIs) are an ongoing health challenge in Samoa and across the Pacific. Samoa consists of four inhabited islands with a relatively small population of approximately 196 000 people. (*1*) The country’s close-knit community dynamic poses unique social barriers to voluntary testing and treatment, and population coverage remains low. (*2*) Samoa has historically reported high rates of chlamydia and gonorrhoea and low rates of HIV and syphilis from cross-sectional antenatal prevalence studies. The first seroprevalence survey in the country, conducted in 2000, found that chlamydia and trichomoniasis infections were common among pregnant women (30.9% and 20.8%, respectively), with a low prevalence of gonorrhoea and syphilis (3.3% and 0.5%, respectively). (*2*) One analysis found that young women aged < 25 years were three times more likely to have an STI than older women. (*3*) Chlamydia is endemic among women, with those < 25 years having greater positivity than those ≥ 25 years (26.1% compared to 11.9%). (*3*) Women who are unmarried or are 18–24 years of age were more likely to test positive for chlamydia. (*4*) Chlamydia infections are also associated with subfertility in Samoan women. (*5*) Maternal and congenital syphilis in Samoa has increased since 2018. (*6*) Untreated maternal syphilis has been associated with fetal loss, neonatal death, premature birth and lower birth weight. (*7*) These results indicate significant health challenges for women of childbearing age.

The 2017 national positivity rates for chlamydia, gonorrhoea and syphilis (24.2%, 5.6% and 1.04%, respectively) are similar to previous prevalence rates. (*8*) This suggests a persistently high prevalence of STIs, particularly chlamydia, for the past decade. Conversely, Samoa has previously reported the lowest rate of syphilis worldwide (0%). (*8*) However, syphilis has increased globally among heterosexuals and men who have sex with men. (*8*) In Samoa, syphilis cases have steadily increased from 26 reported cases in 2015 to 78 cases in 2017. (*6*)

Although prevalence estimates were well established in previous studies, more recent data on sex and age are needed to inform intervention design. Using national surveillance data, this analysis seeks to update the status of the epidemic by comparing positivity rates among sex and age groups from years 2012 and 2017.

## Methods

For a cross-sectional study, the data of all patients in the country who had an STI investigation (routine or suspect) from any provider were analysed for years 2012 (*n* = 18 804) and 2017 (*n* = 48 898) to compare trends in the positive diagnosis of chlamydia, gonorrhoea, hepatitis B and C, and syphilis by age and sex. Due to the low sample size in 2012, syphilis data were compared for 2014 (*n* = 3865) and 2017 (*n* = 11 418). Patients with lost or insufficient specimens were excluded from the sample.

The HIV and STI Surveillance Database, which was used to construct the study sample, is managed by the Samoa Ministry of Health in the capital of Apia. The most complete full-year data sets (2012, 2014 and 2017) were used. Data are comprised of all diagnostic testing (both routine testing and suspect cases in the general population) that was recorded by the National Reference Laboratory (NRL) of Tupua Tamasese Meaole (TTM) Hospital, Apia. In 2017, all national diagnostic technologies were centrally housed in the NRL. All specimens collected in the country are processed by the NRL and recorded in the database. The assays used in the national testing algorithm include polymerase chain reaction (PCR) for chlamydia and gonorrhoea, rapid plasma reagin (RPR) and *Treponema pallidum* haemagglutination (TPHA) tests for syphilis, hepatitis B surface antigen (HBsAg) tests for hepatitis B, the hepatitis C antibody (anti-HCV) test for hepatitis C. Clinicians collect and send specimens to NRL or refer the patient directly to the NRL for collection. Once specimens are processed, the requesting clinician and relevant authorities are notified, and the reporting is collated into the database.

We used Epi Info software (version 7.2.2.6) to estimate odds ratios and χ^2^ test (χ^2)^ to analyse trends (extended Mantel-Haenzsel). Odds ratios are suitable estimations of the prevalence of a disease when infections are less than 10%. (*9*, *10*) Samoa’s syphilis positivity rates ranged from 0.13% in 2012 to 1.04% as of July 2018. (*11*) Cases were compared based on sex (comparing males to females), age (comparing all other age groups to those aged 15–19 years) and the type of STI. Since there were only 10 cases in 2012, data for syphilis were taken from 2014 when there were sufficient cases (*n* = 18) by age to run the analysis without violating the assumption of the extended Mantel-Haenzsel tests (80% of cells have more than five cases). Additionally, syphilis age groups were adjusted from five-year to 10-year age groups (< 45 and  >  45) for sample size, while other STI age groups are compared to those < 30 years and  >  30+ years.

### Sample

The majority of tests come from routine testing (76.0% in 2012 and 78.7% in 2017, **Annex**). This includes testing of women attending antenatal care services, immigration screening for Samoan nationals and blood donation screening. Females represent more total tests than males (71.5% compared to 28.5% in 2012, and 56.7% versus 43.3% in 2017, respectively). There were 7861 tests in 2012 and 11 152 in 2017 in individuals < 30. Testing increased for individuals age  >  30 years (6428 tests in 2012 and 15 145 in 2017). Overall, STI positivity has decreased in the youth age group (15–24 years), from 11.9% in 2012 to 3% in 2017, and in antenatal care testing from 9.5% in 2012 to 1.1% in 2017. Chlamydia was the most common infection (27.9% in 2012 and 22.9% in 2017), with fewer tests in 2017 compared to 2012 (2207 to 4951, respectively). Gonorrhoea is the second most common infection (6.3% in 2012 and 10.0% in 2017), with more tests in recent years (2201 in 2017 compared to 208 in 2012). Hepatitis cases are low, with 2.6% positive rate in 2012 and 2.4% in 2017 for hepatitis B, and 1.1% in 2012 and 0.1% positive for hepatitis C. Testing for hepatitis B and C was higher in 2017 (9780 tests in 2012 and 22 312 in 2017). In 2014, there were 18 cases of syphilis from 3865 tests, and in 2017 there were 90 cases from 11 418 tests.

### Results

Positivity for STIs varied based on age and sex (**Table 1**). Chlamydia and gonorrhoea positivity rates were higher in the age group 15–19 years. In 2012, relative to those aged 15–19 years, those aged 20–24, 25–29 and  >  30 years had significantly lower odds of testing positive for chlamydia (unadjusted odds ratio of 0.80, 0.58 and 0.32, respectively). In 2017, relative to those aged 15–19 years, those aged 25–29 years and  >  30 years had significantly lower odds of testing positive (unadjusted odds ratio of 0.55 and 0.27, respectively). No significant results were observed for sex in 2012 and 2017.

**Table 1:** Odds of chlamydia, gonorrhoea, and hepatitis B and C infections by age and sex, 2012 and 201

-	Chlamydia		Gonorrhoea		Hepatitis B		Hepatitis C	
2012	OR (95% CI)^C^	*p*-value	OR (95% CI)^C^	*p*-value	OR (95% CI)^C^	*p*-value	OR (95% CI)^C^	*p*-value
Sex^a^								
Male	1.05 (0.742–1.48)	0.79	15.73 (3.37–73.42)	0.00*	2.37 (1.83–3.07)	0.00*	9.31 (1.27–68.44)	0.01*
Female	1	-	1	-	1	-	1	-

Age group^b^								
15–19	1	-	1	-	1	-	1	-
20–24	0.80 (0.67–0.96)	0.01*	1.84 (0.19–17.71)	0.58	1.75 (0.77–3.97)	0.18	0.44 (0.07–2.67)	0.36
25–29	0.58 (0.47–0.70)	0.00*	0.97 (0.08–11.48)	0.98	3.86 (1.74–8.56)	0.00*	0.57 (0.09–3.43)	0.53
> 30	0.32	0.00*	1.21 (0.10–14.43)	0.88	5.54 (2.57–11.92)	0.00*	1.28 (0.29–5.58)	0.74

2017	OR (95% CI)^C^	*p*-value	OR (95% CI)^C^	*p*-value	OR (95% CI)^C^	*p*-value	OR (95% CI)^C^	*p*-value
Sex								
Male	0.91 (0.65–1.26)	0.55	7.10 (5.13–9.82)	0.00*	2.48 (1.95–3.17)	0.00*	0.67 (0.21–2.12)	0.50
Female	1	-	1	-	1	-	1	-

Age Group								
15–19	1	-	1	-	1	-	-	-
20–24	0.78 (0.52–1.15)	0.21	0.27 (0.13–0.56)	0.00*	2.56 (0.77–8.49)	0.11	-	-
25–29	0.55 (0.36–0.82)	0.00*	0.55 (0.29–1.04)	0.06	4.35 (1.34–14.12)	0.01*	-	-
> 30	0.27 (0.17–0.41)	0.00*	0.43 (0.23–0.82)	0.01*	11.82 (3.77–37.05)	0.00*	-	-

^a^ reporting only cases where sex is known

^b^ reporting only cases where age is known

**^c^ extended Mantel-Haenszel** χ**^2^ for trend, two-tailed sig.**

* significant at 0.05 level

- insufficient cases for analysis

CI = confidence interval

OR = odds ratio

For gonorrhoea, compared with females, males had greater odds of testing positive in both 2012 and 2017 (unadjusted odds ratio of 15.7 and 7.1, respectively). In 2012, positive tests did not differ significantly by age. In 2017, relative to those aged 15–19 years, those aged 20–24 years and those aged  >  30 years had significantly lower odds of testing positive for gonorrhoea (unadjusted odds ratio of 0.27 and 0.55, respectively).

For hepatitis B, compared with females, males had greater odds of testing positive in both 2012 and 2017 (unadjusted odds ratio of 2.4 and 2.5, respectively). In both 2012 and 2017, relative to those aged 15–19 years, those aged 25–29 and  >  30 years had significantly greater odds of testing positive (2012: unadjusted odds ratio of 3.9 and 5.5, respectively; 2017: unadjusted odds ratio of 4.3 and 11.8, respectively).

For hepatitis C, compared with females, males had significantly greater odds of testing positive in 2012, but not in 2017 (unadjusted odds ratio in 2012 of 9.3 and in 2017 of 0.67). In 2012, with respect to age, there was no difference in test positivity.

For syphilis, compared with females, males had lower odds of testing positive in both 2014 and 2017 (unadjusted odds ratio of 0.28 and 0.63, respectively) (**Table 2**). In 2014, relative to those aged under 45 years, those  >  45 years were 50-fold more likely to test positive. In 2017, compared with those under 45 years, those aged  >  45 years were 14-fold more likely to test positive. For 2017, there were sufficient data to compare smaller age groups. Compared to those aged 15–24 years, all older age groups had greater odds of a positive syphilis test (unadjusted odds ratio of 3.1 for those aged 35–44 years and 25.6 for those aged  >  45 years).

**Table 2:** Odds of syphilis infection by age and sex, 2014 and 2017

-	Positive	Negative	OR (95%CI)^C^	*p-*value
2014				
Sex^a^				
**Male**	6	476	0.28 (0.11–076)	0.01*
**Female**	12	3371	1	-

Age (years)^b^				
**15–24**	0	1582	-	-
**25–34**	4	1492	-	-
**35–44**	1	486	-	-
** > 45**	9	128	-	-
** < 45^d^**	5	3560	1	-
** > 45**	9	128	50.06 (16.54–151.51)	0.00*

2017				
Sex^a^				
**Male**	30	4934	0.63 (0.40–0.97)	0.04*
**Female**	60	6190	1	-

Age (years)^b^				
**15–24**	7	3437	1	-
**25–34**	16	4081	1.93 (0.79–4.68)	0.14
**35–44**	12	1892	3.11 (1.22–7.92)	0.01*
** > 45**	55	1055	25.60 (11.62–56.38)	0.00*
** < 45^d^**	35	9410	1	-
** > 45**	55	1055	14.02 (9.13–21.52)	0.00*

^a^ reporting only cases where sex is known

^b^ reporting only cases where age is known

**^c^ extended Mantel-Haenszel** χ**^2^ for trend, two-tailed sig.**

^d^ insufficient data for age groups 15–44, all < 45 were compared to 45+ in 2014

* significant at 0.05 level.

CI = confidence interval

OR = odds ratio

## Discussion

This analysis finds similar positivity rates to seroprevalence estimations, and it provides gender and age trends in positivity. As expected, young people generally had greater odds of testing positive for chlamydia (high prevalence in both 2012 and 2017) and gonorrhoea, while older age groups were significantly more likely to be positive for hepatitis B and C. This is a common trend as older age groups have longer windows of exposure to viral infections and have had more routine screening over their lifetime, while bacterial infections are more episodic and frequent with young people. Older individuals may also have lower vaccine coverage than younger ones for hepatitis B virus and hepatitis C virus. However, older age groups had greater odds of testing positive for syphilis.

The high positivity rates for chlamydia in 2012 and 2017, in particular, are of great concern. This is due to: 1) the lack of population interventions to reduce chlamydia prevalence; 2) diminishing prioritization of STIs; and 3) the social stigma surrounding sex and STIs that deters individuals from seeking health services and makes delivering comprehensive sexual health education to young people difficult. In a small and close-knit population, seeking services anonymously for taboo conditions is almost impossible and can result in social consequences. (*12*)

It is also unusual for syphilis to be more common in older age groups. We suggest this anomaly may be due to untreated syphilis cases being detected in late tertiary-stage years after an initial infection, when advanced symptoms resurface. Reports indicate a significant increase in infections between 2015 and 2016. (*11*) The odds ratio of syphilis infections was higher in those age  >  45 years both in 2014 and 2017. In addition to older generations being diagnosed in late stages, these results could illuminate a health system-level gap in case detection for syphilis.

From a cultural perspective, older individuals in Samoa are also afforded more social authority, allowing them more agency to freely pursue sexual activity and attend health services. Sexual activity in younger individuals, especially those not married, is considered a more serious taboo and is socially discouraged. (*12*) Additionally, older generations had comparatively less access to sexual health education and prevention. All of these factors would increase the odds of infection in older populations.

In Samoa, government-level STI prevention has focused on young people aged 15–24 years as a high-risk group with the largest burden of infection. (*11*) This study confirms such trends for chlamydia and gonorrhoea. However, syphilis interventions may require age-specific strategies for older populations. These results highlight the need to design age-specific interventions for case detection and prevention in older populations.

### Limitations

The HIV and STI Surveillance Database at the Ministry of Health captures results of all diagnostic tests nationally, with the exception of rapid screening kits. Therefore, the data represent all patients (public, private, routine and suspect) in the country accessing clinical services who received a diagnostic test, excluding screened cases not referred for diagnostics and symptomatic cases treated presumptively. There is selection bias from suspect cases referred to testing by clinicians. Additional studies are needed to estimate the true population prevalence of populations that do not attend services. Information on the stage of infection, exposure and result per assay is not captured.

Each case in the database represents an episode of STI infection, with the possibility of a single patient having multiple episodes. However, anecdotal observations from the Ministry of Health indicate that patients often delay seeking testing and care until infections enter an advanced stage. For instance, only 52% of children under age 5 years with a fever were brought to a health facility for care or advice. (*13*) This late attendance suggests it may be unlikely for patients to report to services more than once a year for STIs.

To obtain a sufficient sample size for syphilis, data were taken from 2014, and age groups were recategorized into 10-year age groups before running the calculations by age and sex, which resulted in larger confidence intervals as the age group categories were less distinct from each other. Additionally, the positivity for TPHA/RPR may indicate chronic yaws infections in older adults, as opposed to syphilis; however, yaws was declared eliminated in the 1960s. (*14*)

## Conclusion

The unexpectedly higher odds of syphilis positivity among adults aged  >  45 years compared to 15–24 years, as well as the persistently high rates of chlamydia infections between 2012 and 2017, highlight a need to re-strategize the promotion and delivery of STI testing and care services in Samoa. Prevention services and strategies should be age-specific, with further assessment of the different barriers for older and younger patients.
